# Intraocular epidermal growth factor concentration, axial length, and high axial myopia

**DOI:** 10.1007/s00417-021-05200-5

**Published:** 2021-05-29

**Authors:** Jost B. Jonas, Li Dong, Shi Da Chen, Michael Neumaier, Peter Findeisen, Songhomitra Panda-Jonas, Rahul A. Jonas

**Affiliations:** 1 Department of Ophthalmology, Medical Faculty Mannheim, Heidelberg University, Mannheim, Germany; 2 Institute of Molecular and Clinical Ophthalmology Basel, Basel, Switzerland; 3 Beijing Tongren Eye Center, Beijing Key Laboratory of Intraocular Tumor Diagnosis and Treatment, Beijing Ophthalmology & Visual Sciences Key Lab, Medical Artificial Intelligence Research and Verification Key Laboratory of the Ministry of Industry and Information Technology, Beijing Tongren Hospital, Capital Medical University, Beijing, China; 4 Fundus Disease Center, Zhongshan Ophthalmic Center, Sun Yat-sen University, Guangzhou, China; 5 Privatpraxis Prof Jonas und Dr Panda-Jonas, Heidelberg, Germany; 6 Institute of Clinical and Scientific Ophthalmology and Acupuncture Jonas & Panda, Heidelberg, Germany; 7 Department of Ophthalmology, Faculty of Medicine and University Hospital Cologne, University of Cologne, Cologne, Germany

**Keywords:** Epidermal growth factor, Myopia, Axial elongation, Myopic maculopathy

## Abstract

**Purpose:**

Various molecules such as dopamine have been found to be associated with axial elongation in experimental studies. Here, we examined whether intraocular EGF is associated with axial length in myopic patients.

**Methods:**

The hospital-based investigation included patients of European descent without optic nerve, retinal, or macular diseases except for myopic maculopathy. Using aqueous humor samples collected during surgery, the EGF concentration was examined applying a cytometric bead array. High myopia was defined by an axial length of ≥ 27.0 mm.

**Results:**

The study included a non-highly myopic group of 11 patients (mean age, 72.9 ± 10.8 years; mean axial length, 24.3 ± 1.1 mm) and a highly myopic group of three patients (age, 81.11 ± 12.3 years; axial length, 29.5 ± 1.3 mm), with one of them having pathologic myopic maculopathy. In multivariable linear regression analysis, higher EGF concentration was correlated with the highly myopic versus non-highly myopic group (beta, 1.24; non-standardized correlation coefficient B, 6.24; 95% confidence interval (CI), 0.10,12.4;*P* = 0.047) after adjusting for axial length. The amount of intraocular EGF was significantly higher in the highly myopic group than in the non-highly myopic group (89.1 ± 40.8 pg versus 34.1 ± 13.2 pg; *P* = 0.005), and it was highest in the eye with myopic maculopathy (135 pg).

**Conclusions:**

The intraocular amount of EGF is higher in highly myopic versus non-highly myopic eyes.

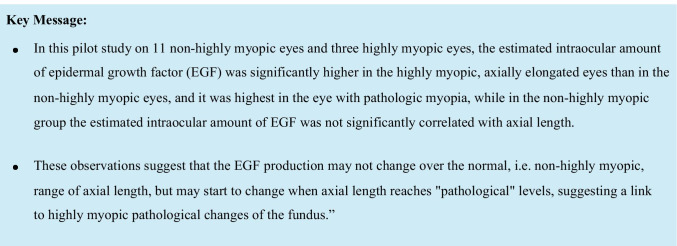



## Introduction

The mechanism underlying the process of axial elongation in axial myopia, in particular in high axial myopia, has yet remained unclear [1, 2]. In histomorphometric studies of eyes of patients aged 3 + years, longer axial length was associated with a retinal thinning and a decreased density of retinal pigment epithelium cells (RPE) in the midperipheral region of the globe, and with a choroidal and scleral thinning most pronounced at the posterior pole [3–7]. In contrast, retinal thickness and RPE density in the macular region and thickness of Bruch’s membrane (BM) in any region of the eye were not correlated with axial length [3, 8–10]. These anatomical observations did not allow drawing firm conclusions about which tissue or tissue layer, the sclera or any other tissue layer such Bruch´s membrane, and by the same token, which “messenger” molecule is primarily involved in the elongation of the globe [11, 12]. Experimental studies on chicken and guinea pigs revealed that various molecules if applied intraocularly or topically had an effect on an externally induced elongation of the optical axis. The list of these molecules includes dopamine and its agonists and antagonists, atropine, transforming growth factor beta (TGF-ß), fibroblast growth factor, hepatocyte growth factor, insulin-like growth factor, and amphiregulin, and other epidermal growth factor (EGF) family members [13–32]. To further elucidate the potential association of EGF and its family members with the process of myopic axial elongation in humans, we undertook this study to examine the intraocular concentration of EGF in the aqueous humor of non-highly patients and patients with high myopia.

## Methods

The clinical interventional study included a group of patients with age-related cataract who consecutively underwent routine cataract surgery, for whom aqueous humor samples had been collected during surgery, and in whose aqueous humor samples the EGF concentration had been measured. The Medical Ethics Committee II of the Medical Faculty Mannheim of the Ruprecht-Karls University Heidelberg approved the study protocol and had waived the necessity to obtain the written consent of the individual patients. All patients were treated at the same institution. Inclusion criterion was the absence of any retinal or optic nerve disease except for myopic maculopathy. Additionally, the volume of the collected aqueous humor had to be at least 100 µL. Intraocular pressure had to be within the normal range of 10 to 21 mmHg.

All patients underwent an ophthalmologic examination including refractometry, applanation tonometry, and slit lamp assisted biomicroscopy of the anterior segment and posterior segment of the eye. Axial length was measured by partial coherence laser interferometry. We defined high myopia by an axial length of ≥ 27.0 mm. The aqueous humor samples were collected at the beginning of the cataract surgery after disinfecting the periorbital skin, lid margins and conjunctiva, draping of the patients, and inserting a lid speculum. The aqueous humor was collected through a temporal paracentesis, before the routine cataract surgery was continued. The aqueous humor samples were deeply frozen in liquid nitrogen within 10 min after collection. The paracentesis was routinely performed to create a temporal access to the anterior chamber for bimanual maneuvering of the lens nucleus and cortex during surgery. The technique has been described previously [33, 34].

The aqueous humor samples were analyzed using the Luminex xMAP suspension array technology (Luminex Co., Austin, Texas, USA). The aqueous humor samples (50 µL) were used undiluted and incubated overnight. The kit was run according to the manufacturer’s instructions. Standard curves for the growth factor (in duplicate) were generated by using the reference EGF concentrations supplied in this kit. All incubation steps were performed at room temperature and in the dark to protect the beads from light. Samples were read on the Luminex xMAP system. Control samples were included in all runs. The detection limit for 0.61 pg/mL with a dynamic range up to 10,000 pg/mL.

The statistical analysis was performed using a commercially available statistical software package (SPSS for Windows, V. 25.0, IBM-SPSS, Chicago, IL). In a first step, we calculated the means of the main outcome parameter (i.e., EGF concentration) and assessed associations of the EGF concentration with other parameters such as age, gender, and axial length. Differences between the highly myopic group and the non-highly myopic group in gender were assessed by Fisher’s exact test and differences in age, intraocular EGF concentration, and intraocular amount of EGF by the Mann–Whitney’s *U* test. For the assessment of a potential association between the EGF-concentration and axial length, we performed a linear regression analysis, in a univariate and multivariable manner. Based on the ocular volume, we additionally calculated the intraocular amount of EGF and assessed its relationship with axial length. The intraocular EGF amount was determined by multiplying the EGF concentration with the estimated ocular volume, which was calculated based on axial length and the formula of a sphere. In the linear regression analyses, we determined the standardized regression coefficient beta, the non-standardized regression coefficient B, and the 95% confidence interval (CI) of B. A *P* value of < 0.05 was considered to be statistically significant.

## Results

The study included a non-highly myopic group of patients and a highly myopic group of patients. The non-highly myopic group consisted of 11 patients (9 men, 3 women) with a mean age of 72.9 ± 10.8 years (median, 70.2 years; range, 57.7–88.5 years) and a mean axial length of 24.3 ± 1.1 mm (median, 24.2 mm; range, 23.01, 26.85 mm). The highly myopic group included three patients (1 man, 2 women) with a mean age of 81.1 ± 12.3 years (median, 87.0 years; range, 67.1–89.3 years) and a mean axial length of 29.5 ± 1.3 mm (median, 30.00 mm; range, 28.10–30.50 mm). One of the three highly myopic patients (axial length: 30.5 mm) had pathologic myopic maculopathy. The non-highly myopic group and the highly myopic group did not differ significantly in age (*P* = 1.00) and sex (*P* = 0.51).

**Fig. 1:**
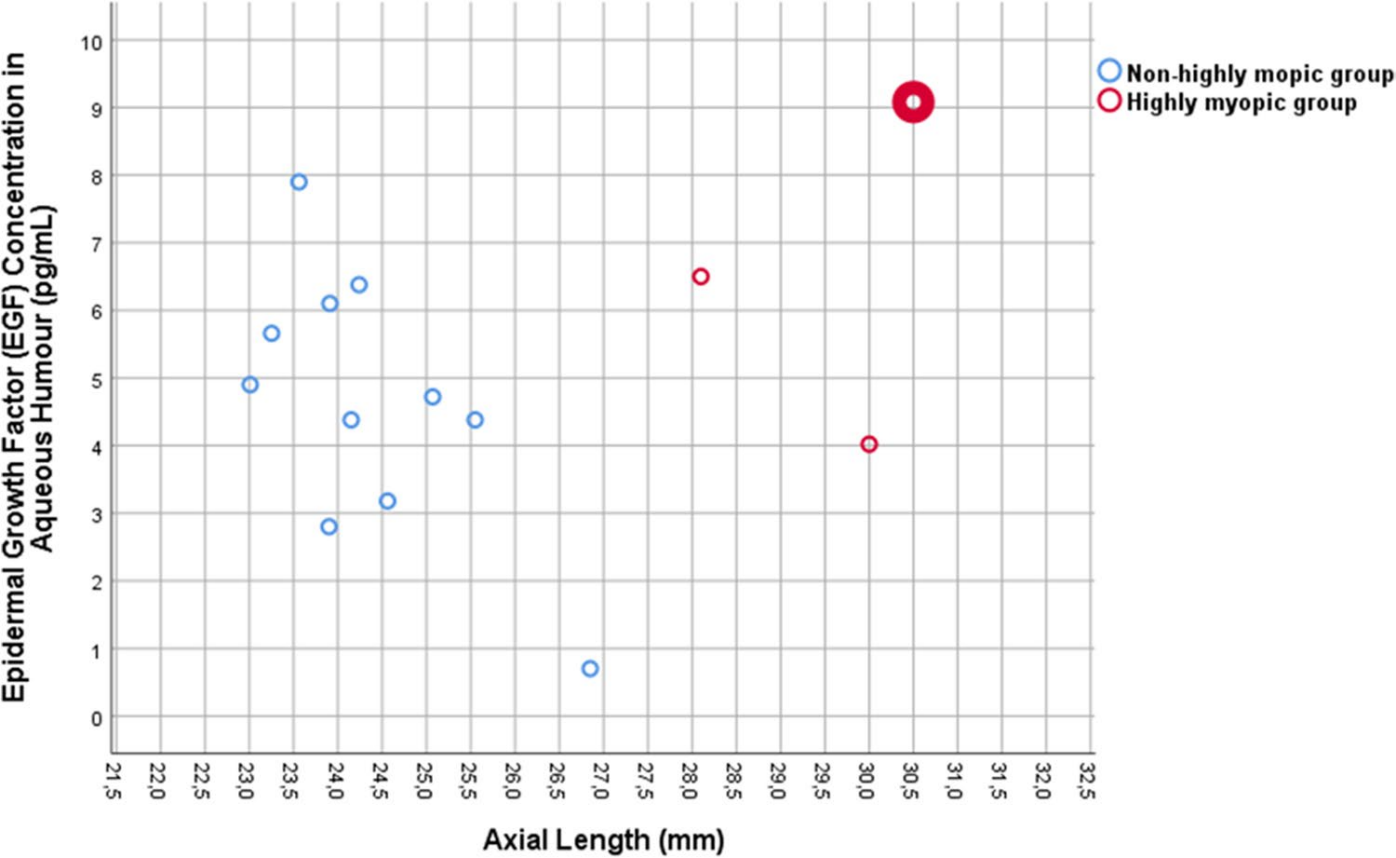
Concentration of epidermal growth factor (EGF) in aqueous humor samples in relationship to axial length. The upper right, red, enlarged point represents the eye with pathologic myopic maculopathy

The mean EGF concentration was 5.05 ± 2.14 pg/mL in the total study population. The mean intraocular EGF concentration was lower, however not significantly lower, in the non-highly myopic group than in the highly myopic group (4.65 ± 1.95 pg/mL (median, 4.72 pg/mL; range, 0.70–7.90 pg/mL) versus 6.53 ± 2.53 pg/mL (median, 6.50 pg/mL; range, 4.02–9.08 pg); *P* = 0.29) (Fig. 1). The EGF concentration was highest in the highly myopic eye with myopic maculopathy (Fig. 1). The EGF concentration was not significantly associated with age (*P* = 0.67) and gender (*P* = 0.35).

In a multivariable linear regression analysis, with the EGF concentration as the dependent variable, a higher EGF concentration was correlated with the highly myopic group versus the non-highly myopic group (beta, 1.24; B, 6.24; 95%CI, 0.10, 12.4; *P* = 0.047) after adjusting for axial length.

**Fig. 2:**
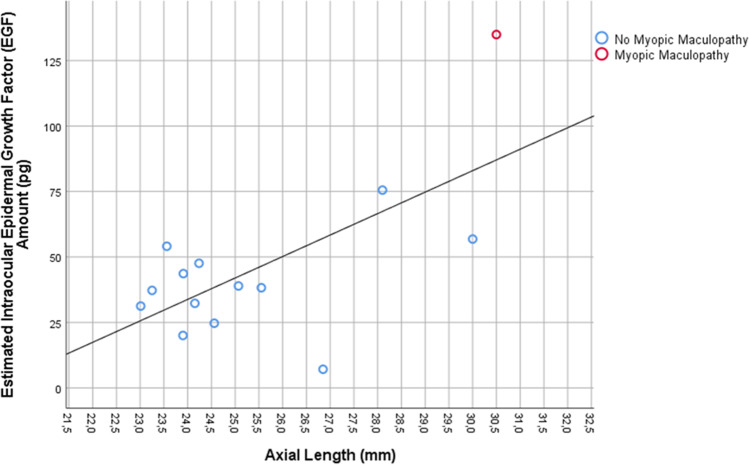
Graph showing the distribution of the estimated intraocular epidermal growth factor (EGF) amount and axial length

In a further step of the analysis, we estimated the ocular volume based on the axial length and using the formula of a sphere. It resulted in a mean ocular volume of 8.9 ± 2.7 mL (median, 7.6 mL; range, 6.5 to 14.9 mL). We then calculated the intraocular amount of EGF by multiplying the EGF concentration with the ocular volume and arrived at a mean EGF amount of 45.9 ± 30.6 pg. The intraocular EGF amount was significantly higher in the highly myopic group than in the non-highly myopic group (89.1 ± 40.8 pg versus 34.1 ± 13.2 pg; *P* = 0.005 (Mann–Whitney test)), and it was highest in the eye with myopic maculopathy (135 pg). In the total study population, the intraocular EGF amount increased significantly with axial length (beta, 0.66; B, 8.19; 95%CI, 2.29, 14.1; *P* = 0.01), while in the non-highly myopic group, the association was not statistically significant and showed a tendency towards a decrease with longer axial length (beta, − 0.51; *P* = 0.11) (Fig. 2).

## Discussion

In our hospital-based study, the intraocular amount of EGF as measured in aqueous humor samples and calculated based on the estimated intraocular volume was significantly (*P* = 0.005) higher in the highly myopic group than in the non-highly myopic group, and it was highest in the eye with myopic maculopathy.

The findings obtained in our study cannot directly be compared with results of other investigations, since the intraocular EGF concentration has not been measured yet in highly myopic eyes. The observations made in a recent study measuring the intraocular concentration of amphiregulin as an EGF family member in myopic eyes remained inconclusive [35]. The retrospective study included 38 eyes with an axial length ranging between 22.4 and 32.4 mm, among them 12 highly myopic eyes without myopic macular degeneration and 12 highly myopic eyes with myopic macular degeneration but without neovascularization. Out of the 38 eyes included into the study, 19 eyes had amphiregulin levels below the limit of detection. In particular, since there were only 4 non-highly myopic individuals with detectable intraocular amphiregulin levels, it was not possible to assess a potential association between the intraocular amphiregulin concentration and axial length in the non-highly myopic group as basis for the comparison of the non-highly myopic group with the highly myopic group.

As shown in Fig. 1, the intraocular EGF concentrations appeared to decrease with longer axial length in the non-highly myopic group. This finding may have been due to two factors, one of them a dilution effect in the eyes with longer axial length and subsequently larger volumes of the vitreous cavity and the anterior chamber. The second factor may perhaps have been a lower viscosity of the vitreous body in axially elongated, myopic eyes, potentially leading to a faster turn-over of intraocular molecules, as has also been suggested by a shorter intraocular availability of intravitreally applied drugs in vitrectomized eyes as compared to eyes with vitreous body [36]. Interestingly, a similar observation of a declining concentration with longer axial length has been made for the intraocular concentration of vascular endothelial growth factor (VEGF) [33, 34, 37]. The finding in our study that the estimated intraocular EGF amount was statistically independent of axial length in the non-highly myopic group supports the notion of a dilution effect as the cause for the declining EGF concentration in the non-highly myopic group (Fig. 2). The statistically non-significant tendency of a slight decrease in the estimated intraocular EGF amount with longer axial length in the non-highly myopic group might have been due to a faster turn-over of the molecule in the myopic eyes with a more fluid vitreous body.

The findings that the intraocular amount of EGF in the non-highly myopic group was not significantly correlated with axial length, that it was significantly higher in the highly myopic eyes as compared to the non-highly myopic eyes, and that it was highest in the eye with myopic maculopathy suggests that the EGF production may not change over the non-highly myopic range of axial length, but may start to increase in the range of high axial myopia. It may suggest a link between the elevated intraocular amount of EGF and pathological changes of the highly myopic fundus.

When the results of our study are discussed, its limitations should be taken into account. First, the number of the patients included into the study, in particular the number of highly myopic patients, was relatively low, so that the present study can only be considered to be a pilot study. Despite of the relatively small number of patients included into the study, the differences between the study groups were statistically significant. Second, the concentration of EGF was measured in aqueous humour samples in which the concentration of growth factors and cytokines is lower than in the intravitreal compartment situation [38]. Obtaining vitreous samples in the patients was however not possible, since it would have necessitated an intravitreal intervention. Also, if vitrectomized eyes had been included in the study, an aspiration of fluid from the vitreous cavity in these eyes would not have been feasible since it would also have unnecessarily extended the surgery. Third, due to its cross-sectional design, our study cannot give information on causal relationships as examined in a longitudinal study.

In conclusion, the intraocular amount of EGF was significantly higher in highly myopic, axially elongated eyes than in non-highly myopic eyes, while in the non-highly myopic group, it was not significantly correlated with axial length. These observations suggest that the EGF production may not change over the normal, i.e., non-highly myopic, range of axial length, but may start to change when axial length reaches “pathological” levels, suggesting a link to highly myopic pathological changes of the fundus.

## Data Availability

All data will be made available upon request.

## Code availability

The statistical analysis was performed using a commercially available statistical software package (SPSS for Windows, V. 25.0, IBM-SPSS, Chicago, IL).

## References

[1] Morgan IG, Ohno-Matsui K, Saw SM (2012). Lancet.

[2] Baird PN, Saw SM, Lanca C, Guggenheim JA, Smith EL, Zhou X, Ohno-Matsui K, Wu PC, Sankaridurg P, Chia A, Rosman M, Lamoureux EL, Man R, He M (2020). Myopia. Nat Rev Dis Primers.

[3] Jonas JB, Ohno-Matsui K, Holbach L, Panda-Jonas S (2017). Retinal pigment epithelium cell density in relationship to axial length in human eyes. Acta Ophthalmol.

[4] Jonas JB, Xu L, Wei WB, Pan Z, Yang H, Holbach L, Panda-Jonas S, Wang YX (2016). Retinal thickness and axial length. Invest Ophthalmol Vis Sci.

[5] Shen L, You QS, Xu X, Gao F, Zhang Z, Li B, Jonas JB (2016). Scleral and choroidal volume in relation to axial length in infants with retinoblastoma versus adults with malignant melanomas or end-stage glaucoma. Graefes Arch Clin Exp Ophthalmol.

[6] Heine L (1899). Beiträge zur Anatomie des myopischen Auges. Arch Augenheilk.

[7] Vurgese S, Panda-Jonas S, Jonas JB (2012). Sclera thickness in human globes and its relations to age, axial length and glaucoma. PLoS ONE.

[8] Jonas JB, Holbach L, Panda-Jonas S (2014). Bruch´s membrane thickness in high myopia. Acta Ophthalmol.

[9] Bai HX, Mao Y, Shen L, Xu XL, Gao F, Zhang ZB, Li B, Jonas JB (2017). Bruch´s membrane thickness in relationship to axial length. PLoS ONE.

[10] Dong L, Shi XH, Kang YK, Wei WB, Wang YX, Xu XL, Gao F, Jonas JB (2019). Bruch’s membrane thickness and retinal pigment epithelium cell density in experimental axial elongation. Sci Rep.

[11] Jonas JB, Ohno-Matsui K, Jiang WJ, Panda-Jonas S (2017). Bruch membrane and the mechanism of myopization. A new theory Retina.

[12] Jonas JB, Ohno-Matsui K, Panda-Jonas S (2019). Myopia: Anatomic changes and consequences for its etiology. Asia Pac J Ophthalmol (Phila).

[13] Gao Q, Liu Q, Ma P, Zhong X, Wu J, Ge J (2006). Effects of direct intravitreal dopamine injections on the development of lid-suture induced myopia in rabbits. Graefes Arch Clin Exp Ophthalmol.

[14] McCarthy CS, Megaw P, Devadas M, Morgan IG (2007). Dopaminergic agents affect the ability of brief periods of normal vision to prevent form-deprivation myopia. Exp Eye Res.

[15] Mao J, Liu S, Qin W, Li F, Wu X, Tan Q (2010). Levodopa inhibits the development of form-deprivation myopia in guinea pigs. Optom VisSci.

[16] Jiang L, Long K, Schaeffel F, Zhou X, Zheng Y, Ying H, Lu F, Stell WK, Qu J (2014). Effects of dopaminergic agents on progression of naturally occurring myopia in albino guinea pigs (Cavia porcellus). Invest Ophthalmol Vis Sci.

[17] Yen MY, Liu JH, Kao SC, Shiao CH (1989). Comparison of the effect of atropine and cyclopentolate on myopia. Ann Ophthalmol.

[18] Barathi VA, Weon SR, Beuerman RW (2009). Expression of muscarinic receptors in human and mouse sclera and their role in the regulation of scleral fibroblasts proliferation. Mol Vis.

[19] Chua WH, Balakrishnan V, Chan YH, Tong L, Ling Y, Quah BL, Tan D (2006). Atropine for the treatment of childhood myopia. Ophthalmology.

[20] Flitcroft DI (2012). The complex interactions of retinal, optical and environmental factors in myopia aetiology. Prog Retin Eye Res.

[21] Mao J, Liu S, Fu C (2016). Citicoline retards myopia progression following form deprivation in guinea pigs. Exp Biol Med (Maywood).

[22] Rohrer B, Stell WK (1994). Basic fibroblast growth factor (bFGF) and transforming growth factor beta (TGF-beta) act as stop and go signals to modulate postnatal ocular growth in the chick. Exp Eye Res.

[23] Seko Y, Shimokawa H, Tokoro T (1995). Expression of bFGF and TGF-beta 2 in experimental myopia in chicks. Invest Ophthalmol Vis Sci.

[24] McBrien NA (2013). Regulation of scleral metabolism in myopia and the role of transforming growth factor-beta. Exp Eye Res.

[25] Li XJ, Yang XP, Wan GM, Wang YY, Zhang JS (2014). Effects of hepatocyte growth factor on MMP-2 expression in scleral fibroblasts from a guinea pig myopia model. Int J Ophthalmol.

[26] Li XJ, Yang XP, Wan GM, Wang YY, Zhang JS (2013) Expression of hepatocyte growth factor and its receptor c-Met in lens-induced myopia in guinea pigs. Chin Med J (Engl) 126:4524–4527 24286418

[27] Cheng T, Wang J, Xiong S, Zhang B, Li Q, Xu X, He X (2020). Association of IGF1 single-nucleotide polymorphisms with myopia in Chinese children. PeerJ.

[28] Jia Y, Hu DN, Zhou J (2014). Human aqueous humor levels of TGF- β2: relationship with axial length. Biomed Res Int.

[29] Chen BY, Wang CY, Chen WY, Ma JX (2013). Altered TGF-β2 and bFGF expression in scleral desmocytes from an experimentally-induced myopia guinea pig model. Graefes Arch Clin Exp Ophthalmol.

[30] Jiang WJ, Song HX, Li SY, Guo B, Wu JF, Li GP, Guo DD, Shi DL, Bi HS, Jonas JB (2017). Amphiregulin antibody and reduction of axial elongation in experimental myopia. EBioMedicine.

[31] Dong L, Shi XH, Kang YK, Wei WB, Wang YX, Xu XL, Gao F, Yuan LH, Zhen J, Jiang WJ, Jonas JB (2019). Amphiregulin and ocular axial length. Acta Ophthalmol.

[32] Dong L, Shi XH, Li YF, Jiang X, Wang YX, Lan YJ, Wu HAT, Jonas JB, Wei WB (2020). Blockade of epidermal growth factor and its receptor and axial elongation in experimental myopia. FASEB J.

[33] Jonas JB, Tao Y, Neumaier M, Findeisen P (2010). VEGF and refractive error. Ophthalmology.

[34] Jonas JB, Tao Y, Neumaier M, Findeisen P (2012). Cytokine concentration in aqueous humour of eyes with exudative age-related macular degeneration. Acta Ophthalmol.

[35] Wong CW, Yanagi Y, Tsai ASH, Shihabuddeen WA, Cheung N, Lee SY, Jonas JB, Cheung CMG (2019). Correlation of axial length and myopic macular degeneration to levels of molecular factors in the aqueous. Sci Rep.

[36] Chin HS, Park TS, Moon YS, Oh JH (2005). Difference in clearance of intravitreal triamcinolone acetonide between vitrectomized and nonvitrectomized eyes. Retina.

[37] Zhu D, Yang DY, Guo YY, Zheng YF, Li JL, Wang B, Tao Y, Jonas JB (2015). Intracameral interleukin 1β, 6, 8, 10, 12p, tumor necrosis factor α and vascular endothelial growth factor and axial length in patients with cataract. PLoS ONE.

[38] Kamppeter BA, Cej A, Jonas JB (2008). Intraocular concentration of triamcinolone acetonide after intravitreal injection in the rabbit eye. Ophthalmology.

